# Endocrine complications in patients with thalassaemia major

**DOI:** 10.1186/1687-9856-2013-S1-P55

**Published:** 2013-10-03

**Authors:** Zhe Meng, Liyang Liang, Lina Zhang, Liping Hou, Zulin Liu, Xiangyang Luo, Jianpei Fang, Zhanwen He, Dongfang Li

**Affiliations:** 1 Department of Pediatrics, Sun Yat-Sen Memorial Hospital, Sun Yat-Sen University, GuangZhou City, China

## 

Thalassemia major is an inherited hemoglobin disorder characterized by chronic anemia and iron overload due to transfusion therapy and gastrointestinal absorption. Iron overload causes severe endocrine complications in patients with multi-transfused thalassaemia major. Endocrine complications includes short stature, acquired hypothyroidism and hypoparathyroidism, hypogonadism, glucose intolerance and diabetes mellitus.

## Aim

The main objective of this study is to determine the prevalence of prominent thalassemia complications.

## Methods

Eighty-seven patients entered the study in our hospital. The patients have a mean age of 10.5 (range, 5-16) years. Physicians collected demographic and anthropometric data and the history of therapies as well as menstrual histories. Patients have been examined to determine their pubertal status. Serum levels of ferritin, glucose, insulin, A1c, cortisol, ACTH,calcium, phosphate, PTH were measured. Thyroid function was assessed by T3, T4 and TSH.

## Results

Short stature was seen in 32.2% of our patients. Diabetes was present in 10.3%, Primary hypothyroidism and hypoparathyroidism was present in 6.9% and 1.15% of the patients. Hypocalcemia was 9.2%. Cortisol and ACTH were normal. About 10.3 % of patients had more than one endocrine complication with mean serum ferritin of 3125 micrograms/lit.

## Conclusion

High prevalence of endocrine complications among our thalassemics signifies the importance of more detailed studies along with therapeutic.

